# Male knowledge, attitude and practice and partner pregnancy among Chinese unmarried youth

**DOI:** 10.1371/journal.pone.0214452

**Published:** 2019-03-26

**Authors:** Ruoxi Ding, Chao Guo, Xinming Song, Xiaoying Zheng

**Affiliations:** 1 Institute of Population Research, Peking University, Beijing, China; 2 APEC Health Science Academy, Peking University, Beijing, China; University of Westminster, UNITED KINGDOM

## Abstract

**Background:**

Early pregnancy among unmarried youth is a serious public health challenge. Male youth’s knowledge, attitude and practice (KAP) of sexual and reproductive health (SRH) and its association with the risk of their sexual partners’ pregnancy in China remains unexplored. This study investigates the KAP among Chinese unmarried male youth aged 15–24 years and identifies its association with partner pregnancy using nationally representative data from Survey of Youth Access to Reproductive Health in China (YARHC) in 2009.

**Methods:**

Chi-square tests were applied to explore the prevalence of partner pregnancy by male youth’s KAP of SRH and logistic regression were applied to identify the associations of partner pregnancy with knowledge, attitudes and practice among male youth.

**Results:**

Among 2853 sexually experienced male youth, totally 597 unmarried male youth caused 852 partner pregnancies and the prevalence rate was 20.93%. Lacking the knowledge of contraception access (OR: 0.66, 95% CI: 0.44–0.99) was significantly associated with lower risk of causing partner pregnancy. Contraception discussion before (1.49, 1.04–2.11) or after first sexual intercourse (1.46, 1.11–1.93), not using valid contraception (1.29, 1.03–1.64) and male contraception decision-maker (1.79, 1.41–2.28) were significantly associated with higher risk of causing partner pregnancy.

**Conclusion:**

Our results indicated that male youth’s knowledge and behaviors of sexual and reproductive health were significantly associated with the risk of causing partner pregnancy, and highlighted the importance and need of sexual and reproductive programs targeting male for the prevention of unintended partner pregnancy in China.

## Introduction

Early pregnancy among teenager and unmarried youth is a serious public health challenge in both developed and developing nations. Worldwide, more than 10 million teenage girls from developing countries had unintended pregnancy each year [1], and the proportion of these pregnancy ended in abortion ranged from 17% in Slovakia to 69% in Sweden [2]. In China, more than 20% of female youth become pregnant every year [3], resulted in almost 6 million induced abortion, and the number are still increasing year by year, caused huge physical, psychological and economic burden for individuals, families and society.

A number of previous studies have reported that female youth’s sexual and reproductive health (SRH) knowledge, attitude and behaviors were associated with the risk of unintended pregnancy. Some studies suggested that a lack of contraceptive knowledge and using valid contraceptive method was associated with an elevated risk of unwanted pregnancy [4,5], and the ambivalent attitudes toward pregnancy and abortion may also increase the likelihood of engaging in risky contraceptive practices and the occurrence of unintended pregnancy subsequently [6]. However, globally, especially in Asia and developing countries, little is known on male youth about their KAP of SRH in relation to pregnancy among their female partners.

The 1994 International Conference on Population and Development in Cairo first proposed male’s important responsibility and underlined the communication between men and women on sexual and reproductive behaviors [7]. As sexual partners, male youth should be actively involved in obtaining adequate reproductive health and contraceptive knowledge, information and services to prevent unintended pregnancy and sexually transmitted diseases. In China and many other developing countries, reproductive health services and education were mainly focused on female adolescents and adults because of the traditional values [8]. However, literatures from less developed regions indicated that male’s attitude and behaviors could play a key role on contraceptive decisions due to the common “dominant male-submissive female” relationship in sexual intercourse [9,10]. Thus, male’s KAP of SRH may have significant influence on the risk of unintended pregnancy among their female partners.

With the dramatic social changes and improving access to social media, the sexual attitude of Chinese youth became more indiscreet. And comparing with previous Chinese generations, premarital sexual intercourse are more common, and the age at sexual debut also declined in today’s young people [11]. However, compared with married persons, less attention has been paid to the education of SRH among Chinese unmarried youth [12]. Therefore, the contraceptive knowledge of Chinese unmarried youth was poor comparing with that among their counterparts in the Western society [5], which may affect their sexual attitudes and contraceptive behaviors, resulted in an increased rate of unintended pregnancy and induced abortion.

In China, the country with a large population of youth and the high rate of induced abortion, unmarried male youth’s KAP of SRH and its association with the risk of their sexual partners’ pregnancy need to be explored for gender-inclusive family planning services and contraceptive education. This study aims to investigate the KAP of SRH among Chinese unmarried male youth aged 15–24 years and to identify its association with partner pregnancy using a nationally representative data.

## Methods

### Data source

Data was obtained from the Survey of Youth Access to Reproductive Health in China (YARHC) [13]. This population-based survey was conducted in 2009, aiming to describe the KAP of SRH among Chinese youth and to investigate the accessibility to reproductive health services for youth. The study protocol was approved by the institutional review board of Peking University Health Science Centre. Informed consents were obtained from all respondents, for those under 18 years, their guardians signed the consents.

### Participants and interview procedure

The target population of YARHC survey are unmarried youth aged 15–24 years in mainland China (Tibet, Hong Kong, Macao, and Taiwan were excluded due to geographic, political, economic, and cultural reasons). A total of 579 interviewers were trained using the standard set by the survey expert committee; Before the formal survey, which was conducted between October 20 and November 30 in 2009, preliminary interviews in a pilot study were conducted in May of the same year. Necessary measures including independent environments and anonymity were ensured to protect the privacy of youth, and all respondents were interviewed face-to-face without a third-party present. Sensitive questions related to sexual experience and behaviors in the questionnaire were self-administered and completed by participants themselves. All completed questionnaires were collected in ballot boxes. Additionally, sex was respected in the survey process, with respondents being interviewed by interviewers of the same sex.

### Samples

The probabilistic samples were drawn using probability proportional to size sampling methods via four stages stratified random cluster. And these four stages for school youth, collective household youth and household youth were cities-schools-classes-students, cities-counties-map pieces (divided according to the streets, rivers and so on in the map)-collective household youth and cities-counties-communities-household youth, respectively. With a sampling ratio of 11.4 per 100 000, the final estimated sample size was 22 535 youth, distributed in total of 40 cities and counties from 25 provinces / autonomous regions / municipalities in China. Since sexual and reproductive health is still sensitive topic in China, some of youth refused to participate in our survey. Besides, there was youth unable to contact or no youth aged 15–24 years in the selected household. Thus, the refusal rate was 24.9%. When the targeting household refuse to participate in the survey, the interviewer followed samples substitution principle, taking the neighbor of non-respondents as the replacement with a swing priority from right to left within 5 units. Finally, 22465 questionnaires were collected, among which 22,288 were valid and 11,212 were male. In this study, due to our study design, only male respondents who ever had sexual intercourse were included. Thus, our samples were restricted to 2853 cases.

### Measures

#### Partner pregnancy

In this survey, if a respondent reported the inserting sex behavior, which was referred to one insert the penis into the vagina, then she/he will be identified as sexually experienced. And for sexually experienced male youth, the answer of the question “Have you ever caused any pregnancy of your sexual partner? If yes, how many pregnancies?” were drawn to create the variable of partner pregnancy (0 as no, > = 1 as yes).

#### Socio-demographic characteristics

Age at the time of the survey was defined as a continuous variable; and residence was classified as urban or rural. Youth were also categorized by education level (junior high school and below, high school, or college and above), mother’s education level (junior high school and below, high school, or college and above), employment (working, not working), and annual family income (low(0–30 000CNY) intermediate (30 001–60 000CNY), or high(60 001–2 100 000CNY)).

#### Knowledge, attitude and practice (KAP) of sexual and reproductive health (SRH)

In this study, KAP referred to the knowledge, attitude, and practice related to sexual and reproductive health among male youth. Knowledge was measured by six dimensions, i.e pregnancy knowledge, abortion impact, contraception methods etc. Attitude was measured by four dimension, including attitude toward abortion, premarital sex and sexual education. Practice was measured by five variables, i.e condom use at sexual debut and the most recent sexual encounter, contraception discussion and so on. The details of KAP questions in the questionnaire were shown in S1 Appendix.

### Statistical analysis

Data were cleaned, checked, and analysed using Stata version 14.0. We summarized descriptive data and used Chi-square tests to explore the prevalence of partner pregnancy by the KAP of SRH among male youth. Univariate logistic regression and multivariate logistic regression were applied to explore the associations between KAP and the partner pregnancy (outcome variable) among male youth. Odds Ratios (OR) and 95% confidence intervals (CIs) were calculated. Considering the potential interaction among knowledge, attitude and practice, we employed three independent models in the analysis. A p-value less than 0.05 was considered statistically significant.

## Results

### Participants characteristics and prevalence of partner pregnancy

The study population comprised 2853 sexually experienced male youth aged 15–24 years. The average age of samples was 20.78 ±2.34years. Urban youth accounted for 59.17% (N = 1688) of the samples, and 14.93% (N = 426) of the study samples only have the educational background of junior high school or below. More than half (54.01%, N = 1541) of the sexually experience male youth were working at the time of survey. Totally 597 male youths caused partner pregnancy, the prevalence rate was 20.93%. And 73.24% (N = 624) of these pregnancies ended in induced abortion.

### Prevalence of partner pregnancy among Chinese male youths by knowledge, attitude and practice (KAP) of sexual and reproductive health (SRH)

Among 2853 unmarried male youth, 95.72% could specify at least one type of contraceptive methods. However, only 57.94% of male youth had the correct knowledge of pregnancy probability, and only 54.64% knew that abortion could impact women’s future pregnancy. The prevalence of partner pregnancy was significantly higher among the male youth who had the knowledge of abortion impact or contraception access and those who were able to get condoms when they need (Table 1).

**Table 1:** Prevalence of partner pregnancy among male youth aged 15–24 years by knowledge of SRH.

Knowledge of SRH		N (%)	Partner pregnancy N (%)	P	
Pregnancy knowledge	yes	1 653 (57.94)	358 (21.66)	0.259
	no	1 200 (42.06)	239 (19.92)	
Abortion Impact	yes	1 559 (54.64)	348 (22.32)	0.044
	no	1 294 (45.36)	249 (19.24)	
Contraception methods	yes	2 731 (95.72)	578 (21.16)	0.137
	no	122 (4.28)	19 (15.57)	
Contraception access	yes	2 596 (90.99)	563 (21.69)	0.001
	no	257 (9.01)	34 (13.23)	
Contraception accessibility	yes	2 356 (82.58)	510 (21.65)	0.039
	no	497 (17.42)	87 (17.51)	
Emergency contraception	yes	2 013 (70.56)	429 (21.31)	0.433
	no	840 (29.44)	168 (20.00)	

Among 2853 sexually experienced male youth, 74.94% had cautious attitude toward abortion and 53.24% have “conditional accept in one case” attitude toward male’s premarital sex. There was significant difference of prevalence of partner pregnancy within the responses to premarital sex among male and female, with the significantly higher prevalence of partner pregnancy was observed among those who have acceptable attitudes toward premarital sex among male and female (Table 2).

**Table 2:** Prevalence of partner pregnancy among male youth aged 15–24 years by attitude of SRH.

Attitude of SRH		N(%)	Partner pregnancy		
			N(%)	P	
Towards abortion	cautious	2 138 (74.94)	451 (21.09)		0.701
	unadvised	715 (25.06)	146 (20.42)		
Towards male’s premarital sex	not accept	210 (7.36)	42 (20.00)		<0.001
	conditional accept in one case	2 288 (80.20)	452 (19.76)		
	accept	355 (12.44)	103 (29.01)		
Towards female's premarital sex	not accept	328 (11.50)	65 (19.82)		0.032
	conditional accept in one case	2 285 (80.09)	466 (20.39)		
	accept	240 (8.41)	66 (27.50)		
Towards sexual education	supportive	2 313 (81.07)	485 (20.97)		0.907
	unsupportive	540 (18.93)	112 (20.74)		

Among 2853 sexually experienced male youth, 42.10% used condom at their sexual debut and 28.46% of male youth never discussed contraception with their sexual partner. The prevalence rate of partner pregnancy was significantly higher among male youth in the groups of not using condom at sexual debut (22.82%), discussing contraception with their partner after their first sexual intercourse (23.08%) and not using valid contraception (22.13%). And the prevalence rate of partner pregnancy was lower among male youths who make the contraception decision with their partner together comparing with those with contraception decided only by themselves or only by their partners (Table 3).

**Table 3:** Prevalence of partner pregnancy among male youth aged 15–24 years by Practice of SRH.

Practice of SRH		N(%)	Partner pregnancy	
			N (%)	P
Condom Use at sexual debut	yes	1 201 (42.10)	220 (18.32)	0.004
	no	1 652 (57.90)	377 (22.82)	
Condom Use at the most recent sexual encounter	yes	1 988 (69.68)	404 (20.32)	0.230
	no	865 (30.32)	193 (22.31)	
Contraception discussion	never	812 (28.46)	144 (17.73)	0.009
	before	568 (19.91)	113 (19.89)	
	after	1 473 (51.63)	340 (23.08)	
Using Valid contraception	yes	820 (28.74)	147 (17.93)	0.012
	no	2 033 (71.26)	450 (22.13)	
Contraception decision-maker	together	1 645 (57.66)	313 (19.03)	<0.001
	male	472 (16.54)	136 (28.81)	
	female	374 (13.11)	92 (24.60)	
	unsure	362 (12.69)	56 (15.47)	

Other information about the prevalence of partner pregnancy by the KAP of SRH among male youth was also displayed in Tables 1, 2 and 3.

### Association between partner pregnancy and the KAP of SRH among Chinese male youth

The association between partner pregnancy and the KAP of SRH among sexually experienced male youth are presented in Table 4. The multivariate regression result of Model 1 showed that after adjusting socio-demographic variables, lacking knowledge of abortion impact and contraception accessibility was not associated with lower risk of partner pregnancy. However, lacking the knowledge of contraception access (OR: 0.66, 95% CI: 0.44–0.99) remains significantly associated with a lower risk of causing partner pregnancy among male youth. No significant association was found between attitude and partner pregnancy (Model 2). For practice (Model 3), contraception discussion before (1.49, 1.04–2.11) or after first sexual intercourse (1.46, 1.11–1.93), not using valid contraception (1.29, 1.03–1.64) and male contraception decision-maker (1.79, 1.41–2.28) were significantly associated with a higher risk of causing partner pregnancy among male youth.

**Table 4:** Association between KAP of SRH and partner pregnancy among male youth aged 15–24 years.

KAP of SRH		Univariate Logistic Regression		Multivariate logistic regression^1^	
		OR	95%CI	OR	95%CI
Model 1					
Pregnancy knowledge (reference = yes)	no	0.9	0.75, 1.08	0.92	0.76, 1.11
Abortion impact(reference = yes)	no	0.83	0.63,1.09	0.87	0.72, 1.05
Contraception methods(reference = yes)	no	0.69	0.42,1.13	0.88	0.53, 1.47
Contraception access(reference = yes)	no	0.55**	0.38, 0.80	0.66*	0.44, 0.99
Contraception accessibility(reference = yes)	no	0.77*	0.60,0.99	0.93	0.70, 1.23
Emergency Contraception(reference = yes)	no	0.92	0.76,1.13	1.01	0.82, 1.24
Model 2					
Attitude towards abortion(reference = cautious)	unadvised	0.96	0.78, 1.18	0.92	0.74, 1.14
Attitude towards male's premarital sex (reference = not accept)	conditional accept in one case	0.98	0.69, 1.40	0.89	0.60, 1.34
	accept	1.63*	1.09,2.46	1.49	0.92, 2.41
Attitude towards female's premarital sex (reference = not accept)	conditional accept in one case	1.04	0.78, 1.39	1.12	0.80, 1.56
	accept	1.53*	1.04, 2.27	1.21	0.75, 1.96
Attitude towards sexual education(reference = supportive)	unsupportive	0.99	0.78, 1.24	1.00	0.78, 1.26
Model 3					
Condom Use at first sexual intercourse(reference = yes)	no	1.32**	1.09, 1.59	1.23	0.99, 1.52
Condom Use at last sexual intercourse(reference = yes)	no	1.13	0.93, 1.37	1.08	0.87, 1.34
Contraception discussion(reference = never)	before	1.15	0.88, 1.51	1.49*	1.04, 2.11
	after	1.39**	1.12, 1.73	1.46**	1.11, 1.93
Using valid contraception(reference = yes)	no	1.3*	1.06, 1.60	1.29*	1.03, 1.64
Contraception decision-maker (reference = together)	male	1.72***	1.36, 2.18	1.79***	1.41, 2.28
	female	1.39*	1.06, 1.81	1.3	0.99, 1.70
	unsure	0.78	0.57, 1.06	0.99	0.67, 1.46

^1^Adjusted for age, residence, education level, mother’s education level, employment and annual family income.

^*^P<0.05

**P<0.01

***P<0.001.

## Discussion

This study investigated the KAP of SRH among Chinese unmarried male youth, and its association with partner pregnancy. Our result suggested that a fairly large proportion of male youth lack the necessary knowledge and cautious attitudes for the prevention of partner pregnancy, especially for the knowledge of pregnancy knowledge and abortion impact. Additionally, the percentage of male youth using valid contraception were still quite low (28.74%) comparing with their counterparts in the Western society, such as United States (62%) and France (70%) [14,15], indicating the inadequacy of sexual and reproductive health education for Chinese male youth.

Our results showed that knowing where to get condom was associated with higher risk of causing unintended pregnancy among Chinese male youth. This is inconsistent with the conclusion in previous research [16] on American young women, which suggested that more correct responses on contraceptive knowledge scale was associated with a decreased risk of unintended pregnancy. This could possibly be explained by the lack of early education on sexual and reproductive health among Chinese male youth [17]. Many of them became aware of contraceptive knowledge only after the occurrence of pregnancy among their female partners [18]. Because of the suppression of sexuality in Confucius culture, the attitude towards premarital sex remains highly conservative in Chinese society and most of families. Many youth in China may only learn about the necessary contraceptive information through hospital visits, indicating that the contraceptive education and family planning services in China are lagging behind the current demand of male youth [17].

In this study, after adjusting for socio-demographic variables, male’s attitude was not significantly associated with the risk of partner pregnancy among Chinese unmarried youth. Previous studies [16,19] for both teenagers and adult women suggested that ambivalent attitudes toward pregnancy and abortion, or low motivation to avoid unintended pregnancy was associated with reduced odds of using contraception consistently. Although our result observed no direct association of attitude and subsequent risk of partner pregnancy, the attitude of sexual and reproductive health is a changing indicator, which may not present the actual attitude at the time of occurrence of partner pregnancy. To integrate attitude with practice might provide more valuable information on the association and its underlying mechanisms.

As expected, we found that male youth’s several behaviors were significantly associated with the risk of causing pregnancy among their female partners. First, inconsistent with the previous study on American African adolescent males, in which who discuss contraception with their partner prior to intercourse were more likely to report condom use, our result found that contraception discussion before or after the first sexual intercourse with their partner was associated with higher risk of partner pregnancy. This may suggest that only communication on contraception may be not a valid enough behavior for condom using and prevention of partner pregnancy among Chinese unmarried male youths. Furthermore, the possibility that contraception discussion between couples resulted from the occurrence of pregnancy cannot be excluded, which may further suggest the inadequacy of contraceptive education for youths in China. Secondly, consistently with previous studies in both China [18] and other countries [20], the risk of partner pregnancy was significant lower among male youth who frequently using valid contraceptive methods. Previous research reported that using valid contraception correctly and consistently were found to be effective way to prevent more than 90% of pregnancies [5]. Our result highlights the necessity of contraception promotion among Chinese unmarried youth. Notably, increased risk of pregnancy was also found among the couples whose decision of contraception use was only made by the male. Due to the long-time influence of Chinese traditional culture, “dominant male-submissive female” sexual relationships still exist, especially in less developed region of China, female’s demands in reproductive health was not fully considered [10]. The lower prevalence of partner pregnancy among male youths who make the decision together with their female partner in our result further implies that reproductive health education and family planning services should clearly target male youth to underline gender equality and to establish correct sexual concept.

It is worth mentioning that, in the current study, inconsistency with the established hypothesis was mainly observed in the association between pregnancy and variables with labile characteristics, such as the knowledge and attitude. Further comprehensive research using longitudinal data is warranted to exclude the variation of such indicators. In addition, we also found that some practice, such as contraception decision-making only by male, is possibly resulted from deep-rooted traditional culture, playing vital role on the occurrence of partner pregnancy. Therefore, for further policy implications, government and the public should begin with dynamic and easily-corrected characteristics, such as the improvement on contraceptive knowledge and establishment of correct attitudes among Chinese male youth, and then keep rectifying the negative influence of traditional culture on sexual relationship in a long-term, in order to reduce the occurrence of pregnancy in Chinese unmarried youth effectively.

## Limitation

This study has several limitations. Firstly, the data we used only focused on unmarried male youth, and the KAP of SRH and its association with pregnancy among married male youth could not be observed. Secondly, due to the cross-sectional design, causal relationships between the KAP and the risk of partner pregnancy were not examined, and the results should be interpreted with caution, further research is required. Thirdly, reporting bias may exist in the KAP and the prevalence rate of partner pregnancy since questions related to sexuality remains to be a very sensitive sexual topic in China, especially among youth. Additionally, due to the questionnaire design, other information about the KAP of SRH including the commitment to avoid pregnancy, the satisfaction degree of using condom etc. among Chinese youth cannot be obtained. A more comprehensive general scale is required to investigate the association and pathways between KAP and partner pregnancy among Chinese males.

## Conclusion

This study provided evidences of the inadequacy of sexual and reproductive education among Chinese unmarried youth, and the association between male’s KAP and partner pregnancy among their female partners, filling the gap of existing related literature in developing nations from male perspective. Our findings highlight the importance and need of sexual and reproductive programs targeting male youth for the prevention of unintended partner pregnancy in China. The gender equality in the responsibility of contraceptive behaviors and the correct sexual attitudes of male youth should also be warranted in the future.

## Supporting information

S1 Appendix Showed the details of knowledge, attitude and practice questions of sexual reproductive health in the questionnaire from Survey of Youth Access to Reproductive Health in China (YARHC). (DOCX)
