# Supplementary material for: Male knowledge, attitude and practice and partner pregnancy among Chinese unmarried youth
Source: PLoS One. 2019 Mar 26;14(3):e0214452. doi: 10.1371/journal.pone.0214452 (PMC6435183; doi:10.1371/journal.pone.0214452)
Supplement: S1 Appendix — (DOCX) [file pone.0214452.s001.docx]

**Appendix Table 1 Variables and related questions in YARHC survey to assess the knowledge, attitude and practice(KAP) of sexual and reproductive health (SRH) among Chinese unmarried male youth**

| KAP | Dimension/ variable | Definition | Assessment  method | Questions and options |
| --- | --- | --- | --- | --- |
| Knowledge | Pregnancy knowledge | Correct cognition for pregnancy probability | Yes(1)  No (2, 3) | Please make a judgment on the following statements: A woman may get pregnant once she has sexual intercourse  1.yes  2.false  3.don’t know/ not sure |
|  | Abortion Impact | Correct cognition for abortion impact | Yes(1)  No (2, 3) | Please make a judgment on the following statements: Abortion can’t impact a woman’s future pregnancy  1.false  2.true  3.don’t know/not sure |
|  | Contraception methods | The number of contraception methods that respondents could specify. | Yes(>=1)  No (0) | What contraception method have you heard of (before interviewer’s prompt)?  0. none  1.Oral contraception  2.contraceptive injection  3.condom  4.morning-after pills  5.Coitus interruptus  6.Rhythm method  7.intrauterine device  8.contraceptive cream  9.female sterilization  10.male sterilization |
|  | Contraception access | Knows where to get condom | Yes(1)  No(2) | Do you know where to get condom?  1.yes  2.no |
|  | Contraception accessibility | Be able to get condom | Yes(1)  No(2, 3) | Can you get condom if you need?”  1.yes  2.no  3.don’t know/ not sure |
|  | Emergency Contraception | Knows how to prevent pregnancy if accidently had unprotected sexual intercourse. | Yes(1)  No(2, 3, 4) | Do you know how to prevent pregnancy if you accidently had unprotected sexual intercourse?”  1.use morning-after pills in 72 hours  2.rinsing vagina with water immediately  3.using routine birth control pills immediately  4. others or don’t know) as “no”. |
| Attitude | Toward abortion | Male’s attitude towards abortion | Cautious(1)  Unadvised(2, 3) | Do you agree with the following point:  I never want things like abortion to happen to myself or my sexual partner”,  1.agree  2.disagree  3.not sure |
|  | Attitude Towards Sexual education | Male’s attitude towards sexual education | Supportive(1)  Unsupportive(2) | Do you think the government should provide contraceptive knowledge for unmarried youth?  1.yes  2.no |
|  | Attitude towards male's premarital sex | Male’s attitude towards male’s premarital sex | Not accept(1)  Conditional accept in one case (2,3,5)  Accept(4) | Which one of the following statement do you agree with?  1.a male should never have premarital sex  2.a male could have premarital sex if he’s going to marry his female partner  3.a male could have premarital sex if he is in love with his female partner  4.a male could have premarital sex whether he is in love with his female partner or not  5.other/ unsure |
|  | toward female's premarital sex | Male’s attitude towards female’s premarital sex | Not accept(1)  Conditional accept in one case(2,3,5)  Accept (4) | Which one of the following statement do you agree with?  1.a female should never have premarital sex  2.a female could have premarital sex if she’s going to marry her male partner  3.a female could have premarital sex if she is in love with her male partner  4.a female could have premarital sex whether she’s in love with her male partner or not  5.other/ unsure |
|  | Attitude Towards Sexual education | Male’s attitude towards sexual education | Supportive(1)  Unsupportive(2) | Do you think the government should provide contraceptive knowledge for unmarried youth?  1.yes  2.no |
| Practice | Condom Use at Sexual debut | Used condom at sexual debut | Yes(1)  No (2) | Condom use at first sexual intercourse  1.yes  2.no |
|  | Condom Use at the most recent sexual encounter | Used condom at the most recent sexual encounter | Yes(1)  No (2) | Condom use at the most recent sexual encounter  1.yes  2.no |
|  | Contraception Discussion | Discuss contraception with sexual partner | Never(1)  Before (2)  After (3) | Have you ever discussed contraception with your last girlfriend/boyfriend?”  1.never  2.before the first sexual intercourse with her/him  3.after the first sexual intercourse with her/him |
|  | Using valid contraception | Frequently used contraception is valid | Yes(1, 2, 3)  No(4, 5, 6, 7) | Which contraceptive methods did you use most frequently?  1.condom  2.oral contraceptive  3.contraceptive injection  4.withdrawal  5.rhythm method  6.other methods  7.never use contraceptives |
|  | Contraception decision-maker | The person who decides whether to use contraception | Male(1)  Female(2)  Together(3)  Unsure(4) | who decided to use contraception or not?  1 male (my decision)  2 female (her decision)  3 together(decided together)  4 unsure (not sure). |
